# Enzyme Sequence and Its Relationship to Hyperbaric Stability of Artificial and Natural Fish Lactate Dehydrogenases

**DOI:** 10.1371/journal.pone.0002042

**Published:** 2008-04-30

**Authors:** Amanda A. Brindley, Richard W. Pickersgill, Julian C. Partridge, David J. Dunstan, David M. Hunt, Martin J. Warren

**Affiliations:** 1 Department of Biosciences, University of Kent, Canterbury, Kent, United Kingdom; 2 School of Biological and Chemical Sciences, Queen Mary, University of London, London, United Kingdom; 3 School of Biological Sciences, University of Bristol, Bristol, United Kingdom; 4 Department of Physics, Queen Mary, University of London, London, United Kingdom; 5 Institute of Ophthalmology, University College London, London, United Kingdom; University of Cape Town, South Africa

## Abstract

The cDNAs of lactate dehydrogenase b (LDH-b) from both deep-sea and shallow living fish species, *Corphaenoides armatus* and *Gadus morhua* respectively, have been isolated, sequenced and their encoded products overproduced as recombinant enzymes in *E. coli*. The proteins were characterised in terms of their kinetic and physical properties and their ability to withstand high pressures. Although the two proteins are very similar in terms of their primary structure, only 21 differences at the amino acid level exist between them, the enzyme from the deep-sea species has a significantly increased tolerance to pressure and a higher thermostability. It was possible to investigate whether the changes in the N-terminal or C-terminal regions played a greater role in barophilic adaptation by the construction of two chimeric enzymes by use of a common restriction site within the cDNAs. One of these hybrids was found to have even greater pressure stability than the recombinant enzyme from the deep-living fish species. It was possible to conclude that the major adaptive changes to pressure tolerance must be located in the N-terminal region of the protein. The types of changes that are found and their spatial location within the protein structure are discussed. An analysis of the kinetic parameters of the enzymes suggests that there is clearly a trade off between *K_m_* and *k_cat_* values, which likely reflects the necessity of the deep-sea enzyme to operate at low temperatures.

## Introduction

The physical properties of the deep-sea create a unique environment, which is characterised by high pressures and low temperatures. Hydrostatic pressure increases by approximately 0.1 megaPascal (MPa) (0.1 MPa = 1 bar) for every *ca.* 10 m increase in depth and, since the average depth of the ocean is over 3500 m, the open oceans have an average pressure of around 35 MPa. Similarly, temperature in the deep-sea is generally low, typically in the range 2 to 4°C, necessitating low temperature adaptations, particularly in enzyme systems [1], [2]. These environmental extremes of the deep-sea have required suitable adjustments within living organisms, especially at the molecular level, to allow normal biological processes to operate [1]–[3]. An understanding of these adaptations will provide important insights into the structure/function relationships of proteins, particularly in relation to protein stability and enzyme catalysis under the extremes of environmental stress.

Although the sensitivity to the environment is often a manifestation of a critical feature of the design of biochemical systems [4] surprisingly little is known about the molecular changes that enable proteins to function at high pressure as found, for example, in deep-sea fish. It is clear that such adaptations have taken place, since alterations in the kinetic and thermal properties of proteins from deep-sea fish have been reported [2], [5], [6]. Moreover, it is recognised that pressure-adaptive kinetic properties may be important for establishing species depth zonation patterns in the ocean [7], [8]. However, no molecular explanation exists to account for these adaptations, especially with respect to the effect of pressure on cytosolic proteins. The deepest areas of the oceans have pressures of up to 120 MPa, greater than the pressures known to cause single-chain proteins to undergo pressure-induced denaturation [9]. For oligomeric proteins, dissociation occurs at much lower pressures. This disruption is due to the effect of hydrostatic pressure on hydrophobic and ionic interactions. In one of the few studies undertaken in this research area, proteins of deep-sea fish have been shown to have an increased resistance to thermal denaturation compared to homologous proteins from shallow-water relatives [1], [2], [6], [9]. This increase in thermal stability is thought to be due to the evolution of especially rigid proteins that are able to resist disruption of tertiary and quaternary structure under high pressure [1], [2], [6], [9]. It is predicted that such rigidity may lead to a lack of flexibility around the active site, thereby lowering the activity of the enzyme. However, this cannot always be the case since some barophilic enzymes still show a rate dependency upon pressure [9].

The effect of pressure has also to be considered in light of the fact that deep-sea enzymes also work efficiently at 4°C. Thermal stability of proteins has been mainly studied in thermophilic microorganisms and the overall conclusion from such studies is that the stability is mediated by modulation of the canonical forces such as electrostatic and hydrophobic interactions found in all proteins [10]. The particular way in which this is achieved varies, however, between proteins and does not arise from the presence of particular amino acids or from post-synthetic modifications. The overall effect of these changes appears as an increase in the rigidity of the protein at mesophilic temperatures (10–45°C). In fact, at low temperatures psychrophilic enzymes (cold adapted enzymes) have a 2–4 fold higher catalytic efficiency in comparison to their mesophilic counterparts. It is thought that cold adapted enzymes have weaker intramolecular interactions that produce more flexible molecular edifices capable of performing catalysis at a lower energy cost [11]. There thus appears to be a dichotomy between adaptations for high pressure and those for low temperature: rigidity versus flexibility.

In this investigation we studied the properties of the enzyme lactate dehydrogenase (LDH) (EC 1.1.1.27) in order to gain a greater understanding of the molecular changes that make variants of this enzyme more suitable for catalysis at both high-pressure and low temperatures. Our approach has been to investigate the performance of both recombinant wild type and hybrid LDH-b, the liver isoform of the enzyme, and to investigate the performance of these variants at a range of hydrostatic pressures. Our aim is not so much to identify how the process of natural selection has led to the evolution of enzymes adapted for life in the deep sea, but rather to investigate the relationship between enzyme sequence, catalytic efficiency and stability at high hydrostatic pressures.

## Results

The majority of previous pressure adaptation experiments have been undertaken using LDH, which is a well characterised enzyme, including the first reports [7], [8] that highlighted that enzymes from deep-living organisms may be pressure adapted. In this study we compared the kinetic and stability properties of recombinantly produced LDH-b from two species of fish: the abyssal grenadier *Coryphaenoides armatus* and the Atlantic cod *Gadus morhua*. These represent two related (Gadiform) teleost species from entirely distinct pressure habitats: *C. armatus* typically living below 2000 m depth, though ranging between 300 and 5000 m (1 – 4°C); and *G. morhua* between 50–200 metres (0 – 20°C) [12]. The relatedness of the two species means that any non-adaptive sequence differences are likely to be minimal, allowing the easier detection of pressure-adapted variations.

**Figure 1:**
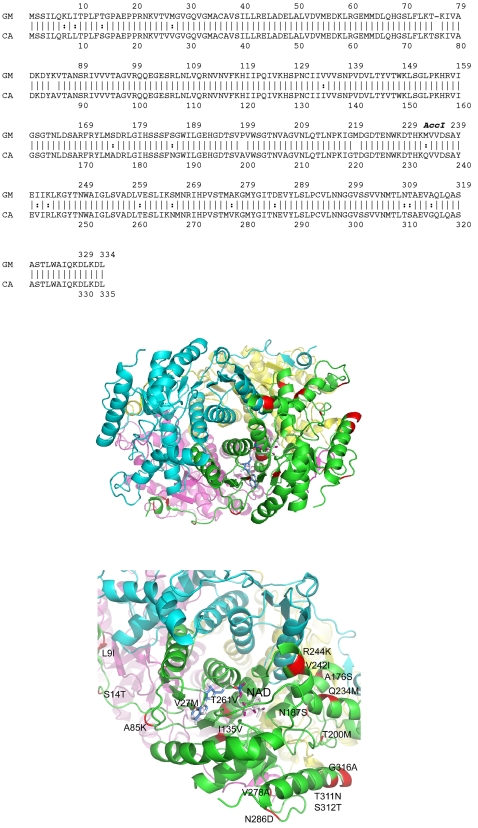
Primary structure of the LDH-b sequences from *C. armatus* and *G. morhua* and their modelling onto the human LDH-b structure. Figure 1a shows the sequence alignment of the *C. armatus* (the abyssal grenadier, or macrourid/rat tail) and *G. morhua* (Atlantic cod) LDH-b. The corresponding relative position of the *Acc*I site is highlighted, from which the hybrid enzymes were generated. Figure 1b and c are cartoon representations of the LDH structure showing the residue substitutions as red splashes on the green subunit. (b) Overview of the tetramer viewed down a 2-fold axis and (c) a close up of the green subunit in similar orientation to that of panel (b) Residues 7, 76, 267, 220 are hidden in this view and the subunits are in different colours: green, cyan, yellow, purple; sites of mutation shown on green subunit in red, and NAD is shown in stick representation. This Figure was made using PyMOL and based on the human LDH structure.

The cDNAs corresponding to LDH-b from both fish were isolated from total liver mRNA extracts and fully sequenced. The cDNA sequences have been deposited in the EMBL nucleotide database; accession numbers are *C. armatus* LDH-b AJ 609232, *G. morhua* LDH-b AJ 609233. Analysis of the cDNAs and the encoded amino acid sequence allowed comparisons with other LDHs in the databases and confirmed the cDNAs as corresponding to the LDH-b paralogue. Phylogenetic analyses of the sequences against other vertebrate LDH sequences, clustered the *C. armatus* and *G. morhua* LDH-b sequences closest together, with other teleosts being next most similar. Alignment of the *G. morhua* and *C. armatus* peptide sequences reveals they share over 93% identity (Figure 1a). There are only 21 different amino acid residues over a total of 335 residues. The most notable difference is deletion of codon 76 from the *G. morhua* sequence. All the known catalytic residues are maintained in both sequences. The sequence data were used to model the structure of the LDH-b onto the known human structure (Figure 1b, c; and see below).

An *Acc*I site present in both species was used to generate hybrid sequences that encoded either the first 236 residues of the *C. armatus* enzyme and the 98 terminal residues of the *G. morhua* LDH (AM) or the first 235 residues of the *G. morhua* enzyme and the terminal 98 residues of the *C. armatus* LDH (MA). The resulting hybrid proteins were used to examine differences in kinetic properties or pressure stability, which could then be assigned to amino acid variances in the N-terminal or C-terminal regions of the protein.

**Figure 2:**
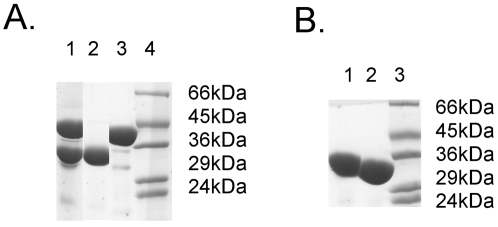
SDS-PAGE of *Gadus morhua* and *Coryphaenoides armatus* ldh-b. Panel A shows the Factor XA cleavage of *Coryphaenoides armatus* ldh-b. Lane 1 is Factor XA overnight digest of the Maltose binding protein-ldh-b fusion protein, lanes 2 is pure *C. armatus* ldh-b, lane 3 is maltose binding protein and lane 4 is Dalton VII marker. Panel B shows the thrombin cleavage of the *Gadus morhua* ldh; lane 1 is His-tagged ldh-b and lane 2 is ldh-b after removal of the His-tag and lane 3 is Dalton VII marker.

The four LDHs were overproduced as recombinant proteins in *E. coli*. The *C. armatus* LDH-b could only be isolated in a soluble form when produced with an N-terminal maltose binding protein (MalE) extension. Similarly, the way in which the AM hybrid was cloned meant that this recombinant protein was also isolated with an N-terminal MalE extension. The *G. morhua* protein was produced with an N-terminal His-tag. The MA fusion protein was also isolated with an N-terminal His-tag. All four overproduced recombinant proteins were purified from *E. coli* extracts by affinity chromatography, followed by cleavage of either the His-tag (*G. morhua* and MA fusion) or the MalE protein (*C. armatus* and AM fusion). This meant that the *G. morhua* and MA hybrid enzymes were thus purified with three extra amino acids at the N-terminus (Gly-Ser-His) whereas the *C. armatus* and AM hybrid enzymes were obtained with 4 extra N-terminal amino acids (Ile, Ser, Glu, Phe). An SDS gel of the purified *G. morhua* and *C. armatus* enzymes is shown in Figure 2. Gel filtration chromatography was employed to remove contaminating His-tag or MalE protein and aggregated protein. It also permitted the determination of the native molecular mass of each LDH-b, all of which were found to be tetramers, which is consistent with all other vertebrate LDHs [13].

### Temperature Stability

Since it has been proposed that molecular adaptations to pressure are similar to adaptations to high temperature [8], the thermal stability of the proteins was investigated. The midpoint of thermal denaturation of proteins was determined by circular dichroism. The proteins were subjected to a thermal melt between 25 °C and 95°C. By assuming that at 25 °C the protein is completely folded and at 95 °C the protein is completely unfolded, the Gibbs free energy of thermal denaturation can be determined and thereby the temperature at which the protein is half way between completely folded and completely unfolded. This then is a measure of the thermal stability of a protein. The midpoint of thermal denaturation for *C. armatus* LDH-b is 74.4±0.4 °C and for *G. morhua* LDH-b 60.6±0.4 °C . The thermal midpoint of denaturation for the MA hybrid was 69.3±0.9 °C, which was very similar to the value observed for the AM protein (70.5±0.9°C) (Table 1).

**Table 1:** Kinetic parameters of the LDH-b enzymes measured in this study.

Protein	K_m_ at 0.1 MPa Pyruvate (µM)	NADH (µM)	k_cat_ at 0.1 MPa (s^−1^)	K_m_ at 75 MPa Pyruvate (µM)	NADH (µM)	k_cat_ at 75 MPa (s^−1^)	Midpoint temp unfold (°C)
C. armatus	160.8 (±4.6)	17.1 (±2.3)	105.0 (±5.2)	170.25 (±5.4)	18.5 (±2.8)	91.5 (±4.8)	74.4 (±0.4)
G. morhua	43.7 (±3.3)	7.4 (±1.2)	40.3 (±3.7)	40.7 (±2.9)	6.7 (±1.7)	19.9 (±2.1)	60.6 (±0.3)
Armatus-Morhua hybrid	31.3 (±4.1)	4.1 (±0.9)	39.3 (±3.4)	38.3 (±3.6)	4.6 (±0.8)	37.1 (±4.1)	70.5 (±0.9)
Morhua-Armatus hybrid	196.3 (±8.7)	34.7 (±2.6)	146.0 (±7.1)	208.0 (±11.4)	31.4 (±3.2)	139.3 (±6.7)	69.3 (±0.1)

### Kinetic Analysis

Kinetic studies were undertaken to determine the apparent *K_m_* values for NADH and pyruvate and the *k*_cat_ values for all the enzymes at both atmospheric pressure and 75 MPa (Table 1). The *C. armatus* enzyme has a *K*_m_ for pyruvate that is significantly higher than that measured for its shallow water cousin. This is in agreement with the observation that the *K_m_* value for pyruvate is higher in LDH enzymes that operate in lower temperature environments, such as those found in the deep sea [8]. The *C. armatus* enzyme also had a higher *k*_cat_ (turnover number), which indicates the overall enzymatic rate. Surprisingly, the AM hybrid had a *k*_cat_ that was significantly reduced in comparison to the *C. armatus* enzyme, even though the major catalytic residues of the *C. armatus* enzyme are located within this hybrid. Conversely, the MA enzyme had activity that was greater than that observed with the *C. armatus* enzyme. These results suggest that the sequence variances located within the C-terminal region of the *C. armatus* LDH-b must contribute to catalysis, even if they are far removed from the active site. Nonetheless, the overall specificity of the enzyme, which is given by the ratio of *k_cat_/K_m_*, shows that all the enzymes have a similar value, indicating that higher turnover numbers are compensated by higher *K_m_* values.

Pressure did not greatly influence any of the kinetic parameters. Major changes were only observed on *k*_cat_ at pressures where the protein was found to denature (see below).

### Effect of pressure on activity and stability of LDH

**Figure 3:**
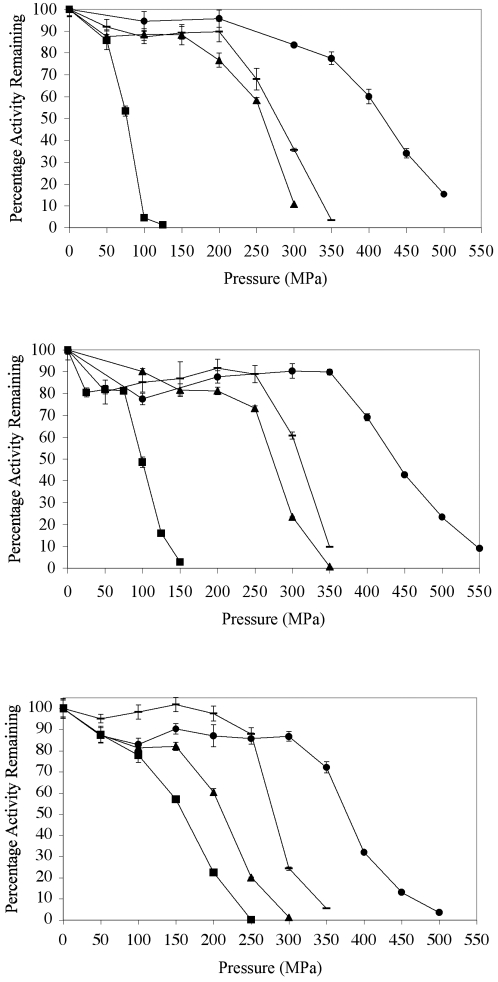
Effect of pressure on the specific activities of all four LDH-bs. Panel A is 1 µg/ml, panel B is 1 µg/ml 250 mM TMAO and panel C is 100 µg/ml. ▪ is GM, ▴ is MA, - is CA and • is AM. Error bars are standard error.

The sensitivity of the recombinant deep and shallow LDHs to pressure was determined by monitoring the effect of pressure on the enzyme at one of two fixed enzyme concentrations: 1 µg per ml and 100 µg per ml. Two protein concentrations were used since it has been reported that protein concentration is a factor in pressure susceptibility [14] although the values chosen are not necessarily indicative of *in vivo* concentrations but reflect ranges used in previous work [14]. The effect of pressure on the activity of *C. armatus* enzyme at 1 µg per ml revealed that pressure sensitivity could be observed around 250 MPa although some activity could still be observed at 350 MPa (Figure 3a). In contrast, the shallow LDH-b equivalent from *G. morhua* at 1 µg per ml showed initial inactivation around 75 MPa with only marginal activity at 100 MPa. Increasing the protein concentration did indeed alter the pressure stability, increasing the stability of both enzymes by a similar extent. At 100 µg/ml the *C. armatus* LDHb displays sensitivity around 300 MPa whereas the *G. morhua* LDH-b displays sensitivity at 150 MPa (Figure 2c). Initial experiments determined the extent of the hybrid's stability at 1 µg/ml in the same manner as the wild type enzymes. The MA hybrid retained 58% activity at 250 MPa and 8 % activity at 300 MPa, and the AM hybrid retained 35% activity at 450 MPa and 10 % activity at 500 MPa. On increasing the protein concentration to 100 µg/ml the pressure stability actually decreased in contrast to the wild type enzymes, this time the MA hybrid only retained 20% activity at 250 MPa and the AM hybrid only retained 13 % activity at 450 MPa. Presumably surface exposed residues in the hybrid enzymes allow some aggregation to take place at higher enzyme concentration under pressure, leading to greater inactivation.

### Effect of osmolytes on protein stability

Osmolytes have been shown to protect proteins from pressure denaturation [15]–[17]. TMAO (250 mM) was added to LDH samples at concentrations of 1 µg/ml and 100 µg/ml prior to the application of pressure (Figure 3b). For the *C. armatus* LDH-b, the addition of TMAO was found to have a significant effect at both the low and high protein concentrations, resulting in the enzyme maintaining about a 20 % higher activity under conditions of pressure to which it was previously sensitive (in effect the pressure sensitive activity profile is moved to the right, Figure 3b). For the *G. morhua* LDH-b, TMAO only had a pronounced effect at the lower protein concentration, resulting in an approximate 50 MPa increase in pressure stability. Surprisingly, there was no major effect of TMAO at the higher enzyme concentration. The hybrid enzymes also demonstrated stabilisation to pressure in the presence of TMAO (Figure 3b).

**Figure 4:**
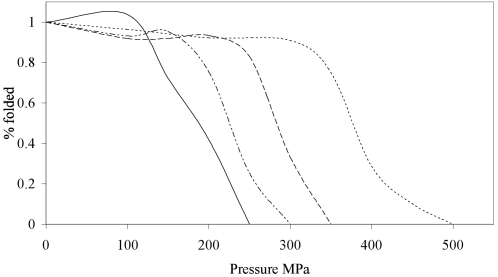
Effect of pressure on protein stability as monitored by CD at 222 nm. The effect of pressure on GM is given by the continuous line, on MA is given by the dashed-dotted line, on CA is given by the dashed line and on AM is given by the dotted line.

Circular dichroism was used to determine a midpoint of pressure denaturation, based on the same principle of the thermal melt. A pressure melt was undertaken up to a pressure where there was no detectable LDH activity at 100 µg/ml. The *C. armatus* LDH gave a midpoint of pressure denaturation of 297 MPa and the *G. morhua* of 177 MPa (Figure 4). Consistent with the activity findings, the MA and AM hybrid enzymes were found to have midpoint pressure denaturation values of 215 and 389 MPa respectively. These values demonstrate that both enzymes have elevated pressure stability with respect to the major protein component from which they are derived.

## Discussion

As predicted, the recombinant LDH-b from the deep-sea fish demonstrates increased tolerance to pressure and the protein also has a higher thermostability [1], [2], [7], [18]. Indeed, the enzyme is substantially more stable than its shallow water orthologue, with resistance increased by over 100 MPa. Previous research had shown that higher protein concentrations also result in greater resistance to pressure, and a similar finding was observed in this study [14]. Similarly, the presence of the osmolyte TMAO resulted in increased protection against pressure [15]–[17]. Our findings thus agree with previous studies that have shown that LDH from deep-sea fish is adapted to withstand high pressures. However, unlike previous studies we have obtained the sequence of the LDH and are thus in a position to try and explain how amino acid changes may influence the barophilic adaptation. More recent studies on α-actin have shown that single amino acid substitutions can alter the pressure sensitivity of a protein although these studies were limited since they were carried out on a mixture of actin isomers [19].

Other work on both LDH and malate dehydrogenase has suggested that high pressure leads to protein unfolding, making the enzymes more susceptible to proteolysis [14], [18]. It has been suggested that pressure first leads to tetramer dissociation and then subunit unfolding [20]. Our analysis by CD spectroscopy clearly points to protein unfolding as the cause for inactivation but it does not support a two-tier disintegration/unfolding process because only one transition is observed. Both the LDH-b from *G. morhua* and *C. armatus* were found to have pressure stabilities much higher than are required in their native environment. However, such stabilities are likely required for continued exposure to pressure, as the pressures applied in this study were for only limited (1 hour) periods.

There are 21 amino acid residue differences between the *C. armatus* LDH-b and the *G. morhua* orthologue. These amino acids are spread equally throughout the protein sequence and represent a mixture of polar and non-polar substitutions. Although it was possible to crystallise the *C. armatus* LDH, the crystals only diffracted poorly and insufficient data were collected to allow a structure determination. Nonetheless, we were able to model the *C. armatus* sequence on to the human LDH structure, which revealed that the amino acid changes occur at both exposed and buried positions within the tertiary structure of the protein and on α-helices, β-strands, and irregular regions of the polypeptide chain (Figure 1b, c). There is no clustering of the mutations. Only one of the substitutions, 241, is at the dimer interface and this change occurs close to the edge of that interface. Two of the substitutions map to the tetramer interface (76 and 267) but are spatially separated. There are no substitutions located within the active site of the enzyme (Figure 1c). Some interesting points can, however, be noted about the substitutions. Going from *G. morhua* to *C. armatus*, three of the substitutions involve the replacements of lysines K7R, K85A and K244R and two of these involve the replacements of lysine with arginine. The substitution of lysine for arginine is known to increase thermostability in glucose isomerase, where mutating out the surface lysines increased stability. This is now known to be a more general effect arguably because the guanido group of arginine is more rigid and can make more hydrogen bonds than the amino group of lysine. The greater rigidity means less entropy is lost on hydrogen bonding contributing to the stability of the protein. Interestingly, these three lysines are all at solvent accessible positions within the LDH structure. Secondly, three methionines are substituted (M27V, M220T and M233Q), suggesting that the side chain may not support pressure stability. The higher pressure may make the sulfur-containing side chain more susceptible to oxidation. Two out of three of these methionines are solvent accessible, M27 would be buried.

The presence of a common restriction site in the cDNAs of the LDHs provided an opportunity to investigate whether all of the amino acid variances between the shallow and deep species LDHs contributed equally to pressure stability.

The two hybrid enzymes, representing the first 235/236 residues of one sequence with the remaining residues of the second protein, might be predicted to show an intermediate stability compared to the native forms, and, indeed, the MA enzyme did show this. However, the AM hybrid displayed an elevated stability to pressure, significantly greater than the native *C. armatus* enzyme. We interpret this result as due to the major adaptive changes to barophilic stability being present in the N-terminal region of the protein. Thus the 12 amino acid changes present in the N-terminus of the protein of the *C. armatus* enzyme are likely to enhance the stability of the protein and protect against high pressure. The N-terminus of the protein constitutes the major part of the Rossmann fold (amino acids 20-162), the protein structural motif found in proteins that bind nucleotides, especially NAD. However, only three changes are actually located within this region. The amino acid variances in the C-terminal region of the *C. armatus* LDH-b appear to enhance the catalytic rate of the enzyme, albeit at the expense of a larger *K_m_* for pyruvate. The pyruvate binding site is made from residues 163–247 and 267–331, and includes 11 of the substitutions that differentiate the two sequences. There is clearly a trade off between a high *K_m_* and high *k_cat_*, which may also reflect the necessity of the enzyme to operate at low temperatures.

In conclusion, we have shown for the first time that molecular changes in the primary structure of a protein allow for enhanced pressure stability. Our modelling shows that amino acid substitutions well away from the active site affect protein stability and enzyme activity. A similar conclusion was reached in a study of LDH-a orthologues from fish that live in different thermal environments [21], where again amino acid substitutions outside the active site provided enhanced thermostability and alterations in kinetic properties. Our study has shown that pressure adaptation, either resulting from natural selection or by artificial manufacture, can be mediated by different amino acid substitutions and we highlight the substitution of lysines and methionines, of which five out of six substitutions occur in the pressure stabilizing N-terminal part of the enzyme, as possible key residues in pressure stability. Thus, changes in the *C. armatus* enzyme in comparison to the *G. morhua* LDH-b make it more stable to pressure, yet the inclusion of the C-terminal region of the *G. morhua* enzyme on the *C. armatus* framework, generating the AM hybrid, makes it even more resistant to high pressure. Therefore, adaptation to pressure is not likely to be caused by similar or identical changes across species (whether organismal of molecular), and we are therefore unlikely to find any consensus change that specifically mediates such a process. This conclusion, whilst based on LDH-b, may have implications and, possibly, applications beyond pure biology and pyruvate metabolism. Enzymes capable of operating at hyperbaric conditions are needed for industrial processes and, while unlikely to be itself of any commercial utility, LDH may provide a good model for the investigation of the fundamental relationship between protein sequence and enzyme function.

## Materials and Methods

### Chemicals and reagents

Most chemicals were purchased from Sigma, Poole, UK. Other materials were provided by the following suppliers: SMART RACE™ cDNA Amplification kit and Advantage 2 PCR System were from CLONTECH, Mountain View, California, USA.; restriction enzymes were from Promega, Chilworth, Southampton, U.K.; chelating Sepharose fast flow resin and gel filtration columns were from GE Healthcare, Little Chalfont, Bucks, U.K.; pET14b was from Novagen, Madison, WI, USA; pMalC2 and amylose resin were from New England Biolabs, Beverly, Maine, USA: all RNA preservation systems were from AMBION, Austin, Texas, USA; RNA extraction kits were from QIAGEN, Crawley, West Sussex, UK; and primers were from Invitrogen, Paisley, UK.

### Fish

*Coryphaenoides armatus* (rat tail) specimens were caught by otter trawl in the Atlantic from the Porcupine Seabight from a depth of 3986 m – 4016 m, September 2000, on RRS *Discovery* Cruise 250. *Gadus morhua* (cod) were caught off the coast of Plymouth at a depth of 30 – 50 m August 2001. Small tissue samples (liver) were removed and stored in RNA Later™ and kept at −20°C.

### Cloning of the LDH cDNA

Total RNA was extracted from the liver samples using the QIAGEN RNAeasy protocol for animal tissues. The RNA was used to make first strand cDNA using the SMART RACE cDNA amplification system, whereby two sets of first strand cDNA are created, 3 and 5 primed cDNA. Degenerate primers were designed from an alignment of all vertebrate LDH-b proteins. Degenerate PCR was carried out on the 3 and 5 primed RACE cDNA and resulted in the isolation of a partial LDH-b cDNA sequence, from which non-degenerate primers were constructed for a further PCR using the Advantage 2 system. This led to the amplification of the full length cDNA, to which primers were designed to clone the cDNA into an expression vector.

The *C. armatus* LDH-b gene was cloned into the pMalC_2_ expression vector to allow the encoded enzyme to be fused at its N-terminal end to the maltose binding protein. The *G. morhua* LDH-b gene was cloned into the pET14b expression vector and the encoded protein was produced as an N-terminally His tagged protein.

### Cloning of Hybrid LDH cDNAs

Generation of an *Armatus-Morhua* fusion; the *C. armatus* LDH-b cDNA in pMalC_2_ was digested with *Eco*RI and *Acc*I, whereas the *G. morhua* LDH-b cDNA in pET14b was digested with *Acc*I and *Hind*III. The two fragments were ligated and cloned into a pMalC_2_ vector cut with *Eco*RI and *Hind*III.

Generation of a *Morhua-Armatus* fusion; the *G. morhua* LDH-b cDNA in pET14b was digested with *Nde*I and *Acc*I, whereas the *C. armatus* LDH-b cDNA in pMalC_2_ was digested with *Acc*I and *Hind*III. After ligation the resulting *Nde*I-*Hind*III fusion was cloned into pET14b that had been cut with *Nde*I and *Hind*III.

### Protein Overproduction and Purification

All four recombinant LDHs were purified from bacterial lysate using affinity chromatography. *C. armatus* (CA) and the A*rmatus-Morhua* (AM) fusion were purified using amylose resin, while the *G. morhua* (GM) and *Morhua-Armatus* (MA) fusion were purified using metal chelating Sepharose according to the manufacturers' instructions. The maltose binding protein was removed after cleavage over night at 4°C with Factor XA (1 unit of Factor XA per 50 µg of fusion protein) and separated from the LDH by gel filtration on a Sephacryl S-300 column. The His-tagged LDH was cleaved overnight at 4°C with thrombin (1 unit of thrombin per 1 mg of His-tagged protein). The His-tag and thrombin were removed by gel filtration on a Superdex 75 HR column.

### Enzyme assays and pressure equipment

The LDH activity was followed by monitoring the decrease in absorbance at 340 nm at 5 °C at atmospheric pressure as described previously [14]. We designed and assembled our own 700 MPa high pressure system. It consists of a cell with optical (sapphire) windows SITEC-Sieber Engineering AG Part No. 740.2006, which fits into a dual-beam optical absorption spectrometer. The cell is surrounded by a water-jacketed encasement to permit temperature-regulated experiments. The sample is contained in a sealed cuvette in the cell in the beam-path between the windows. Pressure is supplied by a capillary feed from a manual screw pump (SITEC 750.01), with water as the pressure fluid. The pump is capable of reaching 700 MPa in less than 2 minutes, and a 50 MPa priming pump is provided to make the higher pressures still easier to reach. The system was chosen to allow very rapid pressure increases to low pressures such as 50 to 100 MPa, so that studies of enzyme kinetics can be carried out within a minimum of time from mixing and loading the reagents into the cell. Pressure was reached and maintained within 1 minute for pressures up to 350 MPa, and within 2 minutes for the highest pressure tested (550 MPa). Temperature was regulated by a circulating water bath and maintained at 5°C (± 0.5°C).

### Pressure studies

Samples were loaded into a 1.4 ml quartz cylinder and a piece of parafilm placed on top and secured with a rubber o-ring, avoiding the presence of air bubbles. Samples were incubated at elevated pressure for 1 h periods, a time frame compatible with other works [14], [17], [18], and then assayed at atmospheric pressure. The pressure range tested was from 25 to 550 MPa.

### Kinetic and structural analysis

Apparent K_m_'s for NADH and pyruvate at both atmospheric pressure and up to 75 MPa were determined at 5°C in 80 mM Tris-HCl (pH7.5), 100 mM KCl and calculated using Delta Graph 4.5. All samples made for CD were at 100 µg/ml in 5 mM sodium phosphate pH 7.2. Protein concentration was determined spectroscopically at 280 nm accounting for the predicted extinction coefficient. SDS-PAGE was used to ensure proteins were >95% pure prior to CD and kinetic analysis. Spectra were run in an Aviv 62DS spectrometer fitted with a peltier temperature controller between 300 nm and 185 nm, with sampling every 0.5 nm and averaging for 3 seconds. The temperature stability of the LDHs was analysed by recording spectra at 5 °C intervals from 25 °C to 95 °C. Pressure stability was analysed by incubating samples at elevated pressure for 1 h, in 25 or 50 MPa intervals depending on the protein and then recording the CD spectra at atmospheric pressure. CD spectra were taken until there was no residual LDH activity detected. CD data were also collected and compared for 5 °C and 25 °C. CD data was deconvoluted using Dichroweb [22]. Sequences, alignments and comparisons were performed with the GCG software package (Genetics Computer Group, Inc. Madison, WI).

## References

[1] Somero GN (1992). Biochemical ecology of deep-sea animals.. Experientia.

[2] Somero GN (1992). Adaptations to high hydrostatic pressure.. Annu Rev Physiol.

[3] Weber G, Drickamer HG (1983). The effect of high pressure upon proteins and other biomolecules.. Q Rev Biophys.

[4] Hochachka PW, Somero GN (2002). Biochemical Apadtation — mechanism and process in physiological evolution.

[5] Siebenaller JF (1984). Structural comparison of lactate dehydrogenase homologs differing in sensitivity to hydrostatic pressure.. Biochim Biophys Acta.

[6] Swezey RR, Somero GN (1982). Polymerization thermodynamics and structural stabilities of skeletal muscle actins from vertebrates adapted to different temperatures and hydrostatic pressures.. Biochemistry.

[7] Siebenaller J, Somero GN (1978). Pressure-adaptive differences in lactate dehydrogenases of congeneric fishes living at different depths.. Science.

[8] Somero GN, Siebenaller JF (1979). Inefficient lactate dehydrogenases of deep-sea fishes.. Nature.

[9] Gross M, Jaenicke R (1994). Proteins under pressure. The influence of high hydrostatic pressure on structure, function and assembly of proteins and protein complexes.. Eur J Biochem.

[10] Scandurra R, Consalvi V, Chiaraluce R, Politi L, Engel PC (1998). Protein thermostability in extremophiles.. Biochimie.

[11] Gerday C, Aittaleb M, Arpigny JL, Baise E, Chessa JP (1997). Psychrophilic enzymes: a thermodynamic challenge.. Biochim Biophys Acta.

[12] Cohen DM, Inada T, Iwamoto T, Scialabba N (1990). Gadiform Fishes of the World (Order Gadiformes) An annotated and illustrated catalogue of cods, hakes, grenadiers and other gadiform fishes known to date.. Food and Agriculture Organisation of the UN (FAO) Fisheries Synopsis.

[13] Read JA, Winter VJ, Eszes CM, Sessions RB, Brady RL (2001). Structural basis for altered activity of M- and H-isozyme forms of human lactate dehydrogenase.. Proteins.

[14] Hennessey JP, Siebenaller JF (1985). Pressure inactivation of tetrameric lactate dehydrogenase homologues of confamilial deep-living fishes.. J Comp Physiol [B].

[15] Yancey PH, Fyfe-Johnson AL, Kelly RH, Walker VP, Aunon MT (2001). Trimethylamine oxide counteracts effects of hydrostatic pressure on proteins of deep-sea teleosts.. J Exp Zool.

[16] Yancey PH, Rhea MD, Kemp KM, Bailey DM (2004). Trimethylamine oxide, betaine and other osmolytes in deep-sea animals: depth trends and effects on enzymes under hydrostatic pressure.. Cell Mol Biol (Noisy-le-grand).

[17] Yancey PH, Siebenaller JF (1999). Trimethylamine oxide stabilizes teleost and mammalian lactate dehydrogenases against inactivation by hydrostatic pressure and trypsinolysis.. J Exp Biol.

[18] Hennessey JP, Siebenaller JF (1987). Pressure-adaptive differences in proteolytic inactivation of M4-lactate dehydrogenase homologues from marine fishes.. J Exp Zool.

[19] Morita T (2003). Structure-based analysis of high pressure adaptation of alpha-actin.. J Biol Chem.

[20] Schmid G, Ludemann HD, Jaenicke R (1979). Dissociation and aggregation of lactic dehydrogenase by high hydrostatic pressure.. Eur J Biochem.

[21] Holland LZ, McFall-Ngai M, Somero GN (1997). Evolution of lactate dehydrogenase-A homologs of barracuda fishes (genus Sphyraena) from different thermal environments: differences in kinetic properties and thermal stability are due to amino acid substitutions outside the active site.. Biochemistry.

[22] Lobley A, Whitmore L, Wallace BA (2002). DICHROWEB: an interactive website for the analysis of protein secondary structure from circular dichroism spectra.. Bioinformatics.

